# Prediction of Asthma Exacerbations in Children by Innovative Exhaled Inflammatory Markers: Results of a Longitudinal Study

**DOI:** 10.1371/journal.pone.0119434

**Published:** 2015-03-23

**Authors:** Dillys van Vliet, Ariel Alonso, Ger Rijkers, Jan Heynens, Philippe Rosias, Jean Muris, Quirijn Jöbsis, Edward Dompeling

**Affiliations:** 1 Department of Pediatric Pulmonology, School for Public Health and Primary Care (CAPHRI), Maastricht University Medical Centre (MUMC+),Maastricht, The Netherlands; 2 Department of Methodology and Statistics, CAPHRI, MUMC+, Maastricht, The Netherlands; 3 Department of Medical Microbiology and Immunology, St Antonius Hospital, Nieuwegein, The Netherlands; 4 Department of Pediatrics, Orbis Medical Center, Sittard, The Netherlands; 5 Department of Family Medicine, CAPHRI, MUMC^+^, Maastricht, The Netherlands; The Hospital for Sick Children and The University of Toronto, CANADA

## Abstract

**Background:**

In asthma management guidelines the primary goal of treatment is asthma control. To date, asthma control, guided by symptoms and lung function, is not optimal in many children and adults. Direct monitoring of airway inflammation in exhaled breath may improve asthma control and reduce the number of exacerbations.

**Aim:**

1) To study the use of fractional exhaled nitric oxide (FeNO) and inflammatory markers in exhaled breath condensate (EBC), in the prediction of asthma exacerbations in a pediatric population. 2) To study the predictive power of these exhaled inflammatory markers combined with clinical parameters.

**Methods:**

96 asthmatic children were included in this one-year prospective observational study, with clinical visits every 2 months. Between visits, daily symptom scores and lung function were recorded using a home monitor. During clinical visits, asthma control and FeNO were assessed. Furthermore, lung function measurements were performed and EBC was collected. Statistical analysis was performed using a test dataset and validation dataset for 1) *conditionally specified models*, receiver operating characteristic-curves (ROC-curves); 2) *k*-nearest neighbors algorithm.

**Results:**

Three conditionally specified predictive models were constructed. Model 1 included inflammatory markers in EBC alone, model 2 included FeNO plus clinical characteristics and the ACQ score, and model 3 included all the predictors used in model 1 and 2. The area under the ROC-curves was estimated as 47%, 54% and 59% for models 1, 2 and 3 respectively. The *k*-nearest neighbors predictive algorithm, using the information of all the variables in model 3, produced correct predictions for 52% of the exacerbations in the validation dataset.

**Conclusion:**

The predictive power of FeNO and inflammatory markers in EBC for prediction of an asthma exacerbation was low, even when combined with clinical characteristics and symptoms. Qualitative improvement of the chemical analysis of EBC may lead to a better non-invasive prediction of asthma exacerbations.

## Introduction

Asthma is the most common chronic inflammatory disorder in children [1]. Worldwide epidemiological studies show that asthma is still not optimally controlled in many children and adults, despite proper pharmacotherapy and emphasis on asthma control in international asthma management guidelines [2–6]. Asthma control is determined by clinical symptoms, lung function, and occurrence of asthma exacerbations [2–4]. Exacerbations are accompanied by loss of quality of life, higher costs due to extra clinical visits and absence from school and work [7].

Currently, management of asthma and titration of treatment is based on the level of asthma control determined by symptoms and lung function [2–4]. However, these measures of asthma control do not give direct insight into the underlying inflammatory process. Measures of airway inflammation such as fractional exhaled nitric oxide (FeNO) and biomarkers in exhaled breath condensate (EBC) reflect airway inflammation in a non-invasive manner. This is in contrast with an invasive procedure such as bronchoscopy with assessment of inflammatory markers in bronchial alveolar lavage or biopsies [8]. These procedures are too invasive for routine and repeated use (in children). It is hypothesized that the management of asthma may improve by monitoring of airway inflammation and by titration of treatment on basis of inflammatory parameters in exhaled breath [9, 10].

In a previous longitudinal study in 40 children with asthma, exacerbations were predicted by Interleukin 5 (IL-5) in EBC and acidity of EBC (Inflammation Asthma Monitoring, FLAME-study) [9]. FeNO is an exhaled marker of inflammation, which reflects eosinophilic airway inflammation [11]. So far, titration of medication based on FeNO has not been associated with significant decrease in the number of exacerbations [11–13]. However, 1 study was underpowered, which may have contributed to this finding. Moreover, the predictive value of FeNO for an asthma exacerbation may improve, when combined with clinical characteristics and other exhaled markers of inflammation.

Before this concept of treating asthmatic children based on these exhaled markers of inflammation can be tested in a clinical randomized controlled trial (RCT), the accuracy of this method should be confirmed in a new population. Therefore, the aims of this study are: 1) To assess the predictive power of FeNO and inflammatory markers in EBC, and their combination for asthma exacerbations in a pediatric population. 2) To assess the predictive power for asthma exacerbations of exhaled markers of inflammation combined with clinical characteristics such as atopy, bronchial hyperresponsiveness, reversibility to a bronchodilator, asthma control, and daily dosage of inhaled corticosteroids (ICS).

## Methods

### Study design and patients

Children between 6 and 18 years old with doctor-diagnosed asthma were recruited for this one year observational cohort study (clinicaltrial.gov NCT 01239238). All children had been treated for asthma at the outpatient clinic of 2 specialized pediatric pulmonology centers for at least 6 months and used inhaled corticosteroids during the year preceding the study. Asthma was defined by the criteria of the Global Initiative for Asthma (GINA) and the guideline of the Dutch Society of Pediatrics as: 1) presence of asthma symptoms and use of ICS during the year preceding the study [2, 4]; 2) reversibility to a β_2-_agonist defined as an increase in FEV_1_ of ≥ 9% of predicted value [4, 14], as described before [9]; and/or 3) presence of bronchial hyperresponsiveness (defined as a > 20% drop in FEV_1_ after the inhalation of histamine with a concentration ≤ 8 mg/ml) [4]. Exclusion criteria were: 1) technical unsatisfactory performance of lung function measurements, 2) presence of cardiac abnormalities, 3) mental retardation, 4) congenital abnormalities or existence of a syndrome, 5) active smoking, or 6) treatment with immunotherapy during the study.

During the study, the investigators were blinded for the study results. The parents received financial compensation for transportation costs to the clinic. The recruitment of patients started in November 2010 and the follow-up ended March 2013.

The Medical Ethical Committee of the Maastricht University Medical Centre approved this study. Informed consent was signed by all parents and by children aged 12 years and older.

### Study parameters and procedures

The primary outcome measure was the occurrence of an exacerbation. Regular clinical visits took place every 2 months. During each clinical visit, the same measurements and sequence of procedures took place. All measurements were performed by 3 trained research nurses. First, children completed the Asthma Control Questionnaires (ACQ). FeNO assessment, EBC collection, and dynamic spirometry were then performed (Table 1). Between clinical visits, daily home monitor assessments were performed by the children and electronic questionnaires were completed by the parents.

**Table 1:** Overview of study parameters.

Visit	0 months	2 months	4 months	6 months	8 months	10 months	12 months
**ACQ**	•	•	•	•	•	•	•
GINA symptom score*	•	•	•	•	•	•	•
**FeNO**	•	•	•	•	•	•	•
**EBC collection**	•	•	•	•	•	•	•
**Dynamic spirometry**	•	•	•	•	•	•	•
**PC**_**20**_ **test**	At the start of the study						
**Registration of exacerbations**	During the entire study						
**Home monitoring** ^†^	During the entire study						

ACQ = Asthma Control Questionnaire, GINA = Global Initiative for Asthma, FeNO = Fractional exhaled Nitric Oxide, EBC = exhaled breath condensate, PC_20_ = histamine bronchial hyperresponsiveness test.

* Symptom score based on GINA criteria was collected during 2 weeks preceding the clinical visit. This score was combined with FEV_1_, to assess asthma control as defined by GINA.

^†^ Home monitoring consisted of daily symptom score plus FEV_1_ measurements.

### Questionnaires

The ACQ was used to assess asthma control at the clinical visits [15]. The cut-off points used for level of asthma control were: ACQ ≤ 0.75 (controlled asthma); 0.75<ACQ ≤1.5 (partly controlled); and ACQ>1.5 (uncontrolled asthma). The ACQ results were not used in the treatment protocol. Treatment (step-up/step-down) was based on asthma control using the GINA-criteria [2]. For this purpose, the GINA-respiratory symptoms during the 2 weeks preceding clinical visits and lung function parameters during clinical visits were used.

### FeNO assessment

FeNO was measured online using a NIOX analyzer (Aerocrine, Solna, Sweden) according to American Thoracic Society/European Respiratory Society (ATS/ERS) criteria [16]. The animation as provided by the manufacturer was used to support children to establish a stable airflow during the maneuver. A standard flow rate of 50 ± 5 ml/sec was required for a correct maneuver.

### EBC collection, storage and chemical analysis

EBC was collected by means of an optimized borosilicate glass tube, cooled by circulating water of 0.7 degrees Celsius, as reported previously [17]. Children breathed for 10 minutes into the tube, through a mouthpiece connected to a two way non-rebreathing (series 1420; Hans Rudolph Inc, Kansas City, USA) valve, which also served as a saliva trap. The child used a nose-clip, breathed tidally and was entertained by a movie or game. Acidity of the condensate was measured without de-aeration, immediately after collection (handheld pH-meter, typePH1000H, and mic-micro-S7 pH sensor, VWR International B.V., NL, Germany). The EBC-samples were separated into aliquots and immediately frozen using dry ice and stored at −80 degrees Celsius. From collection to chemical analysis freeze-thaw cycles were avoided [18]. For chemical analysis 1 sample of at least 60μL was thawed. The levels of interleukin (IL) 1α, IL-5, IL-6, IL-8, IL-13, IL-17 and tumor necrosis factor (TNF) α were determined using a commercially available high sensitivity bead-based flow immunoassays and concentrations were calculated using BioPlex software version 5.1 (Millipore, St Charles, MO, USA). The calibration line consisted of the calibration fluids that were added to the plate according to the manufacturer’s protocol plus 2 sequential dilutions to decrease the lower limit of quantification (LLoQ) of the assay. The median LLoQ’s assessed during analysis of the EBC samples were for IL-1α: 636 fg/ml, IL-5: 13 fg/ml, IL-6: 112 fg/ml, IL-8: 25 fg/ml, IL-13: 30 fg/ml, IL-17: 575 fg/ml and TNFα: 23 fg/ml.

Concentrations of samples were calculated by extrapolation when fluorescence indices were below the LLoQ, yet above background. If outcomes could not be extrapolated, a concentration of 50% of the lowest measured concentration for the specific marker was imputed like described previously [17].

### Dynamic spirometry

Dynamic spirometry was performed by means of the ZAN 100 spirometer, according to ATS/ERS standards (nSpire Health GmbH, Oberthulba, Germany) [19]. The highest value of 3 correctly performed maneuvers was used for analysis. Recorded parameters included: FEV_1_, forced vital capacity (FVC) and maximum expiratory flow at 50% of FVC (MEF_50_), all expressed as a percentage of the predicted value. Subsequently, the patient inhaled 400 μg salbutamol by means of a spacer. After 15 minutes, a second lung function test was performed in order to assess the reversibility to a β_2_-agonist. Patients were instructed to stop short-acting bronchodilators at least 8 hours and long-acting bronchodilators at least 48 hours before testing.

### Bronchial hyperresponsiveness

*Bronchial hyperresponsiveness* was evaluated at the start of the study by a bronchial histamine challenge test [20]. At first, an aerosol of buffered saline was inhaled, followed by aerosols of histamine solutions with doubling concentrations from 0.03 mg/mL to 16mg/mL, at intervals of 5 minutes. After complete inhalation of a solution, the FEV_1_ was measured at 30, 90 and 120 seconds. The percentage decline in FEV_1_ was calculated and the test was stopped when a drop of 20% or more in FEV_1_ occurred, or the highest concentration of 16 mg/mL histamine was administered. The PC_20_ was calculated from a log concentration versus dose response curve. After reaching the threshold, children inhaled 800 microgram salbutamol, followed by 3 Maximal Expiratory Flow Volume maneuvers. 16% of the PC_20_ tests were missing, because of recurrent active airway infections, inability to stop antihistamines or bronchodilators, or calibration errors of the histamine nebulizer.

### Atopy

Atopy was defined by either a positive Phadiatop (Phadia, Uppsala, Sweden) or RAST (Pharmacia, Uppsala, Sweden) or Allergen Skin test. In 3% of the children allergy tests were missing.

### Home monitoring

Daily home monitoring was performed using an AM2+ home monitor (Jaeger, CareFusion, Houten, The Netherlands). Participants were asked to use the home monitor on a daily basis at approximately the same time of the day and were carefully and repeatedly instructed. The AM2+ home monitor is a hand held monitor that can store respiratory symptom scores and lung function measurements. The respiratory symptom score consisted of a short questionnaire including asthma symptoms based on the GINA-criteria for asthma control. The lung function measurements consisted of 3 maneuvers with maximal effort to achieve FEV_1_. The highest FEV_1_ value of each day was selected for each patient. The data were transferred into a secured portal twice a week.

### Definition of asthma exacerbations

Moderate or severe asthma exacerbations were defined according to the latest ATS/ERS criteria [21].

### Asthma treatment protocol during study

Asthma medication was titrated based on GINA asthma level of control, according to GINA guidelines and the guideline of the Pediatric Pulmonology section of the Dutch Society of Pediatrics [2, 4]. In the adjustment of treatment, FeNO values, markers in EBC or home monitor results were not taken into account.

### Sample size calculation

The criterion for sample size calculation was reliable estimation of sensitivity and specificity of the predictive model. We considered a maximum estimation error of sensitivity or specificity of 0.15. The corresponding standard error is 0.075 (Maximal SE = 2*SE; Max SE = 0.15/2 = 0.075). We expected a true risk of exacerbation of 45%, based on the previous FLAME-study [9]. This resulted in inclusion of at least 91 children (SE = sqrt (p*(1-p)/N), where p = true sensitivity or specificity, N = sample size for that group). We assumed a dropout rate of 10% during 1 year follow-up, so we aimed to include at least 100 children.

### Statistical data analysis

All the statistical analyses were carried out using the SAS software package version 9.2. In the explanatory analysis of the data numerical variables were summarized using means and standard deviations (SD), or medians and Inter Quartile Ranges (IQR, i.e. 25^th^–75^th^ percentile). Furthermore, categorical variables were summarized using frequency tables and percentages.

Due to the longitudinal nature of the study, responses from the same patient are naturally correlated. To account for this correlation conditional models were used. Conditional models describe the current outcome of a patient, in a sequence of longitudinal measurements, conditional upon (subsets of) the previous outcomes and covariates [22]. In the present study the probability of an asthma exacerbation between the current and next visit was modeled using 3 conditional models. The first model used the 8 cytokines in EBC (IL-1α, IL-5, IL-6, IL-8, IL-13, IL-17, TNFα), sex, age, trial center and the occurrence (or not) of an exacerbation in the period between the previous and current visit, as covariates. In the second model, FeNO, reversibility to a bronchodilator as increase in FEV_1_% of predicted value, PC_20_, daily dosage of ICS, atopy, ACQ score, sex, age and trial center and the occurrence (or not) of an exacerbation in the period between the previous and current visit were used as covariates. In the third model, the covariates from the first and second model were combined. The models were fitted using the procedure GENMOD in SAS.

For the analysis 11 randomly selected patients (76 measurements) were used to create a validation dataset and measurements (n = 543) of all the remaining patients were included in a training dataset. All predictive models were estimated using the information in the training dataset. Posteriorly the predictive capability of inflammation markers was assessed using the information in the validation dataset. The predictive capability of the models was primarily evaluated using the area under the corresponding ROC-curves. Additionally, the percentage of correct predictions was also assessed using the validation data.

Alternatively, the *k*-nearest neighbors algorithm (KNN) was also used to evaluate the predictive performance of the exhaled markers of inflammation [23]. KNN is a non parametric lazy learning algorithm that uses vectors of predictors or covariates as training samples, each with a class label. The training phase of the algorithm consists only of storing the feature vectors and class labels of the patients in the training dataset. The KNN algorithm was implemented using the SAS procedure DISCRIM.

## Results

### Patients

**Fig 1:**
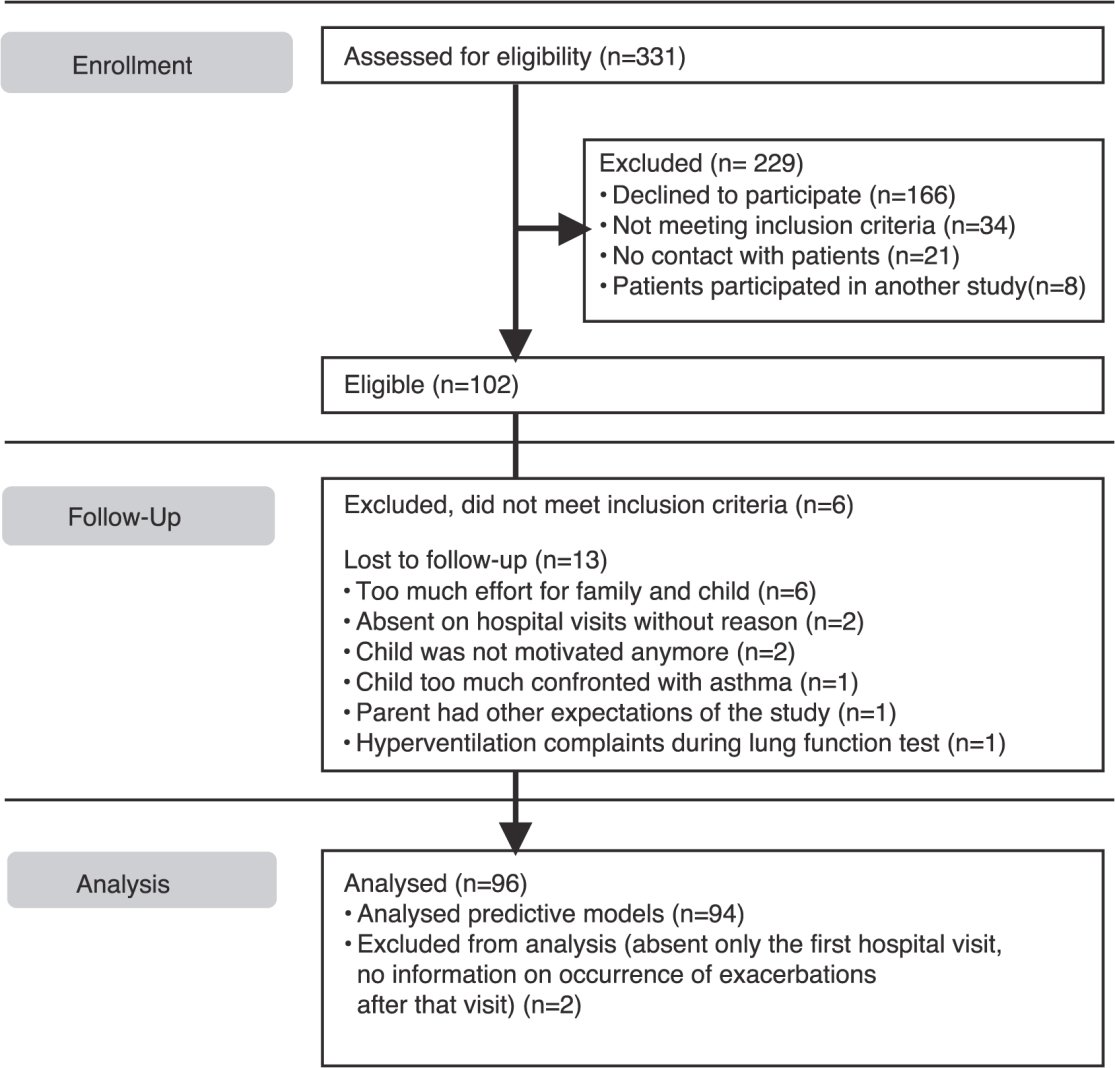
Consort flow diagram of the study.

In the 2 participating hospitals, 331 children with doctor-diagnosed asthma were asked to participate in the study. Of these children, 102 children and parents were willing to participate (Fig. 1). These children were assessed for eligibility based on inclusion criteria. Six of these children were excluded, because they could not perform dynamic spirometry maneuvers correctly (n = 1), did not meet the asthma criteria (n = 4) or had comorbidity with difficulty in distinction of symptoms between the diseases (n = 1).

96 children with asthma started the study. The majority of subjects was atopic and had severe bronchial hyperresponsiveness despite a moderate daily dose of fluticasone (or equivalent) (Table 2). There were no clinically relevant differences in baseline characteristics between the centers. During the study 13 children dropped out. Of these children, 2 were excluded from the statistical models because these children were only present during the first clinical visit and it was unknown whether they experienced an exacerbation in the following 2 months (‘intention to treat analysis’). No protocol deviations occurred.

**Table 2:** Patient characteristics at baseline.

	Total n = 96	Site A n = 51	Site B n = 45
**Sex male, n (%)**	50 (52)	27 (53)	23 (51)
**Mean age [range] in years**	10 [6 – 17]	9 [6 – 17]	10 [7 – 15]
**FEV**_**1**_ **% predicted value, mean ± SD**	96.8 ± 14.2	95.4 ± 15.5	98.4 ± 12.7
**Bronchodilator response, delta FEV**_**1**_ **% predicted value: mean ± SD**	6.6 ± 8.5	6.7 ± 9.8	6.5 ± 7.0
**ICS use, %**	94	90	98
ICS Fluticasone daily dosage or equivalent, mean ± SD*	269 ± 175	295 ± 202	240 ± 134
**ACQ, median [IQR]**	0.6 [0.3–1]	0.5 [0.3–1.0]	0.6 [0.1–1.1]
**FeNO, ppb: median [IQR]**	12.5 [8.0–31.0]	13.0 [8.0–35.0]	12.0 [8.0–27.0]
**PC**_**20**_, **mg/mL: median [IQR] †**	1.2 [0.3–2.9]	1.0 [0.3–2.3]	1.4 [0.4–3.2]
**Atopic, %‡**	76	77	76

Site A = Maastricht, Site B = Sittard, FEV_1_ = forced expiratory volume in one second, SD = Standard Deviation, ICS = Inhaled Corticosteroids, ACQ = Asthma Control Questionnaire, IQR = Inter Quartile Range, PAQLQ = Pediatric Asthma Control Quality of Life Questionnaire, FeNO = Fractional exhaled Nitric Oxide.

* Six children did not use ICS at baseline.

^†^ PC_20_: concentration of histamine inducing a 20% drop in FEV_1_.

^‡^ Atopy is defined as a positive Phadiatop (Phadia, Uppsala, Sweden), or RAST, or a positive allergen skin test.

### Frequency of exacerbations

48% of all 94 children experienced 1or more exacerbations during the study. Of all exacerbations 5 were severe in 5 children and 72 exacerbations were moderate.

### Description of exhaled markers of inflammation

The mean time between collection of EBC and occurrence of an asthma exacerbation was 33 days (standard deviation 17 days). In Table 3, the distribution of concentrations of FeNO, acidity of EBC and EBC inflammatory markers is given. The inflammatory markers in EBC and FeNO exhibited a large variability in concentrations as reflected by the wide ranges and IQR.

**Table 3:** Concentrations of inflammatory markers in EBC and FeNO.

Inflammatory markers	n	Minimum	Maximum	Median	IQR
**pH**	611	3.31	8.80	5.80	5.52, 6.20
IL-1α, fg/ml*	619	0.30	23482.80	98.00	0.30, 483.20
**IL-5, fg/ml**	619	0.05	169.20	2.75	0.05, 19.33
**IL-6, fg/ml**	619	0.10	207.10	1.45	0.10, 21.20
**IL-8, fg/ml**	619	0.01	4508.51	0.01	0.01, 2.30
**IL-13, fg/ml**	619	0.05	393.80	4.43	0.05, 31.30
**IL-17, fg/ml**	619	0.15	1977.10	11.08	0.15, 184.53
**TNFα, fg/ml**	619	0.01	352.10	0.63	0.01, 11.45
**FeNO, ppb**	612	5.00	196.00	15.00	9.00, 27.00

IL = interleukin, TNF = tumor necrosis factor, FeNO = Fractional exhaled Nitric Oxide.

### Predictive power of exhaled marker of inflammation

**Fig 2:**
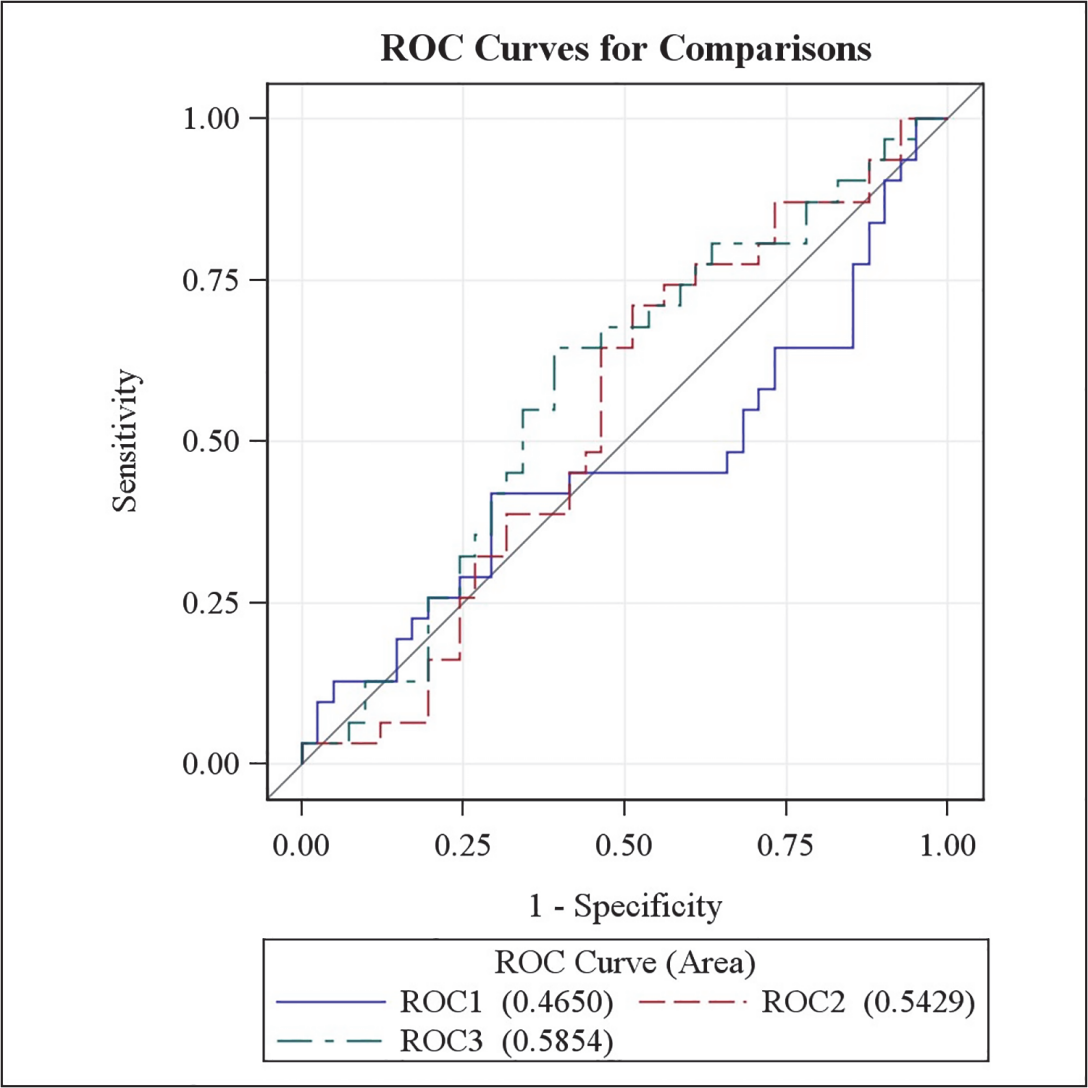
Overview of ROC-curves of 3 predictive models for asthma exacerbations. ROC1: exacerbation prediction model on the basis of the acidity of EBC and inflammatory markers in EBC alone; ROC2: model on the basis of FeNO, reversibility to a bronchodilator as increase in FEV1% of predicted value, PC_20_, daily dosage of ICS; ROC3: model all variables of model 1 and 2.

The results obtained from model 1 clearly illustrate that information on acidity of EBC and inflammatory markers in EBC alone are not suffice to carry out accurate predictions (Fig. 2). The estimated parameters for this model are provided in Table 4. Notice that, although interesting in an explanatory context, the point estimates and p-values are rather irrelevant in a prediction context. More relevant in a prediction scenario is the area under the ROC-curve and for model 1 it was estimated as 47% (Fig. 2). The area under the ROC-curve of model 2, consisting of FeNO and clinical characteristics was estimated as 54% (Fig. 2).

**Table 4:** Performance of acidity of EBC, inflammatory markers in EBC, FeNO, and asthma clinical characteristics in prediction of asthma exacerbation.

Inflammatory markers	estimate	95% CI	p-value
**pH**	0.345	−0.029, 0.718	0.071
**IL-1α**	−0.0004	−0.001, 0.0001	0.1
**IL-5**	0.014	−0.019, 0.047	0.394
**IL-6**	0.002	−0.015, 0.020	0.798
**IL-8**	−0.004	−0.012, 0.003	0.26
**IL-13**	−0.005	−0.018, 0.009	0.488
IL-17	−0.001	−0.002, 0.001	0.359
**TNFα**	−0.001	−0.053, 0.051	0.96
**FeNO**	−0.011	−0.029, 0.007	0.228
**Atopy**	−0.182	−0.985, 0.620	0.656
**PC_20_**	−0.129	−0.347, 0.090	0.248
**Bronchodilator response, delta FEV_1_ % predicted value**	−0.047	−0.104, 0.011	0.111
**daily dosage of ICS**	−0.001	−0.003, 0.001	0.308
**ACQ score**	0.082	−0.424, 0.589	0.751

IL = interleukin, TNF = tumor necrosis factor, FeNO = Fractional exhaled Nitric Oxide, PC_20_ = histamine bronchial hyperresponsiveness test, FEV_1_ = forced expiratory volume in one second, ICS = Inhaled Corticosteroids, ACQ = Asthma Control Questionnaire.

As expected, adding more predictors further improved the predictive performance of the model. For instance, for the most complex model 3 the area under the curve equaled 59% (Fig. 2). Nonetheless, the performance of all models was rather poor and the confidence interval for the area under the curve contained the random guessing value 50% for all the models.

Similar results were obtained when the KNN algorithm was used. Similar to ROC-curves, the most complex model 3 delivered the best prediction when using the KNN algorithm. In fact, model 3 showed an overall correct prediction of 52% in the validation dataset (Table 5).

**Table 5:** KNN- prediction of asthma exacerbation based on acidity of EBC, inflammatory markers in EBC, FeNO, and asthma clinical characteristics.*

Exacerbation	Prediction		
	No n(%)	Yes n(%)	Total n(%)
**No**	24 (33)	17 (24)	41 (57)
**Yes**	17 (24)	14 (19)	31 (43)
**Total**	**41 (57)**	**31 (43)**	**72 (100)**

* KNN algorithm is performed as statistical technique.

## Discussion

In this study we investigated the prediction of asthma exacerbations in children by means of exhaled inflammatory markers (acidity of EBC, inflammatory markers in EBC, FeNO) and clinical asthma characteristics. Collection of EBC was successful in all children. The ability of a combination of acidity of EBC and inflammatory markers in EBC and FeNO to predict asthma exacerbations in children was poor. The prediction improved by the combination of exhaled inflammatory markers and clinical characteristics (reversibility to a bronchodilator, PC_20_, daily dosage of ICS). However, FeNO and clinical characteristics had no additional value on the predictive power of inflammatory markers in EBC. The AUC for the model with all the variables was 59%, which was close to the level of 50% of random guessing. Thus, FeNO and inflammatory markers in EBC assessments every 2 months, were not useful for the prediction of exacerbations in this study.

Neither acidity of EBC, inflammatory markers in EBC, FeNO, nor the combination of these biomarkers was able to predict asthma exacerbations in children. Few studies have focused on the ability of cytokines or chemokines in EBC to predict asthma exacerbations. Therefore, we have compared our results with studies assessing related and/or distinct inflammatory markers in EBC, or cytokines in BAL or sputum of children with asthma exacerbations.

First, we reviewed the studies which assessed the same EBC markers in exacerbations of childhood asthma. Our finding of poor predictive capabilities of cytokines/chemokines in EBC in this study is in contrast to findings of an earlier study[9]. Robroeks et al. reported that IL-5 in and the pH of EBC were predictors for exacerbations in 40 children with asthma in the FLAME-study. There is considerable overlap between the FLAME-study and our study regarding design, measurements and inclusion criteria. However, we were obliged to change the chemical analysis of EBC and have applied another statistical method to build the predictive model, which will be both discussed below. Second, we looked for published data regarding markers of inflammation in EBC and exacerbations in asthma. Baraldi et al. compared cysteinyl leukotrienes (CysLTs) concentrations in EBC at the start of an asthma exacerbation in asthmatic children, before and 5 days after prednisone treatment [24]. Equal to the cytokines in our study, CysLTs are synthesized in the early-phase and late-phase asthmatic reaction. These investigators reported a significant decrease of CysLTs after treatment with prednisone. Debley et al. measured CysLTs in EBC in children during acute asthma exacerbations and 1, 2 and 4 weeks after hospital discharge [25]. As the EBC CysLT concentrations changed little during 1 month follow-up period, their findings suggest that EBC CysLT concentrations do not discriminate between a stable period and an acute exacerbation [25]. In line with CysLTs, hydrogen peroxide, a marker for oxidative stress, was not significantly changed after treatment with bronchodilators or oral steroids compared to during asthma exacerbation [26].

Third, regarding cytokine biomarkers in sputum samples, IL-8 and IL-5 concentrations were assessed in (induced) sputum samples of asthmatic children during and 2 weeks after recovery of an acute asthma exacerbation [27]. In this study IL-5 was only detectable in 29% of the sputum samples collected during asthma exacerbation and no significant change in IL-5 during the course of asthma exacerbation was found [27]. The low detection rate of IL-5 corresponds to our finding that IL-5 has limited power to predict an asthma exacerbation. In contrast, IL-8 concentration was significantly reduced in sputum after recovery of the asthma exacerbation, which was not reflected in our EBC analysis [27].

Fourth, our finding that FeNO could not predict an asthma exacerbation is in line with findings of De Jongste et al. and by a meta-analysis by Petsky et al. [13, 28]. In the study of De Jongste et al. in atopic asthmatics, daily FeNO measurements took place, which limited the time span between FeNO measurements as much as possible [13]. However, even in this case FeNO could not sufficiently predict an asthma exacerbation. In a recently performed RCT, management based on FeNO levels had a positive effect on asthma outcome [29]. Although these researchers found that significant less children in the FeNO group had ≥ 1 exacerbation(s) compared to the control group, no statistically significant difference in exacerbation rate was detected between the groups [29]. Therefore, our data and those of others suggest that FeNO is not an useful predictor for asthma exacerbations in children.

A strength of this study was the focus on a combination of noninvasive inflammatory markers (inflammatory markers in EBC in combination with FeNO) along with clinical parameters to predict asthma exacerbation in children using longitudinal assessments. Another strength was the use of a home monitor for collection of daily symptom scores and lung function (FEV_1_), in order to increase the chance to detect all exacerbations. Although we tried to keep the design and methods in the FLAME-study and the current study comparable, 2 important changes had to be made. First, the assay originally used to assess cytokines in EBC was no longer available. Therefore, we switched to a commercially available kit. Even though the original assay was reliable, switching also implicated that our method could be more easily transferred to other laboratories. Despite being sensitive, we were unable to reproduce the basic findings of the FLAME-study with the commercially available kit [9]. This may indicate that this kit is not suitable for the EBC matrix and more effort has to be made to design assays suitable for EBC.

Second, we switched to another statistical method, namely *conditionally specified models*, which is an advantage of the current study [22]. This type of model has progressed the past years and takes into account the interdependency between repeated measurements within 1 child. In addition, 2 datasets were used for statistical analysis in the current study, namely a training dataset (543 measurements) and a validation dataset (76 measurements), whereas in the former FLAME-study only 1 dataset was analyzed with Cox regression. It is not likely that switching models has affected the results of the current study.

We cannot exclude that we may have found different results if we had implemented shorter time periods between regular visits. The time frame of 2 months was based on the design of the FLAME-study in which we found significant results with 2-monthy intervals. With the observed discrepancies between the studies, these time frames remain a matter of further investigation. In a post hoc analysis based on results of daily FeNO measurements to predict an asthma exacerbation in children, at least 3 to 5 FeNO measurements in the 3 weeks preceding the exacerbation were needed to predict an exacerbation [30]. One single measurement was not sufficient for prediction of an exacerbation. Overall, more work has to be done to determine the optimal time frame between an asthma exacerbation, and increase in parameters of airway inflammation. Therefore, choosing a shorter time between visits may be more successful, but may not be manageable for patients. Even in this study, patients dropped out because clinical visits every 2 months proved too cumbersome.

There are several explanations for the low predictive capabilities of inflammatory markers in EBC in this study. First, as mentioned, we have used commercially available hypersensitive kits to analyze the EBC samples. Despite being sensitive, the detection of cytokines in EBC did not meet expectations, which contributed to the poor predictive properties of the mediators. Second, the exhaled markers of inflammation behaved similarly in a period without exacerbations versus a period before and during an exacerbation. This may be the consequence of the large variability in concentrations of inflammatory markers in EBC. The variability could be influenced by: a) lack of sensitivity of antibodies of the assays used for chemical analysis, or b) lack of assays adjusted for use with EBC as matrix. The ICS daily dosage could not mask possible predictive properties of the inflammatory markers, because this clinical parameter was included in all predictive models.

So far neither assessment of biomarkers in EBC nor FeNO measurements, should be implemented in clinical care to predict an asthma exacerbation. Despite our findings, the non-invasive character of the inflammatory markers and direct measurement of airway inflammation keeps this approach attractive. More (methodological) research is needed 1) to develop EBC adjusted assays; 2) to develop appropriate storage techniques for EBC to avoid negative effects of the EBC matrix on the stability of cytokines (such as low pH). Similar to the dried blood spot storage method, an alternative option may be to absorb and store EBC onto filter paper and avoid negative effects of, for instance proteases [31]. To establish these goals, more collaboration between researchers and industry is necessary and should be stimulated. Beside improvement of assays for inflammatory marker detection in EBC, new information regarding the metabolomics approach of EBC is promising. Metabolomic profiles demonstrated to be specific for severe asthma and to allow adequate discrimination from non-asthma patients [32, 33].

In conclusion, the power of FeNO and inflammatory markers in EBC separately or combined, for prediction of an asthma exacerbation with the current methodology was low. The results from the former FLAME-study could not be reproduced in this external validation study. This may be due to methodological problems associated with storage and analysis of EBC. There is an urgent need for improvement in the chemical analysis of inflammatory markers in EBC.
